# Assessment of Mechanical Detection Thresholds in Healthy Participants and Patients With Neuropathy: A Comparison of OptiHair2 and Aesthesiometer II

**DOI:** 10.1002/ejp.70078

**Published:** 2025-07-17

**Authors:** Jan D. Wandrey, Annika Reinecke, Andrea Westermann, Thomas Lücke, Elena Enax‐Krumova, Christoph Maier, Jan Vollert, Lynn Eitner

**Affiliations:** ^1^ Charité – Universitätsmedizin Berlin, Corporate Member of Freie Universität Berlin and Humboldt‐Universität zu Berlin, Department of Anaesthesiology and Intensive Care Campus Charité Mitte and Campus Virchow‐Klinikum Berlin Germany; ^2^ Berlin Institute of Health at Charité – Universitätsmedizin Berlin, BIH Biomedical Innovation Academy, BIH Charité Digital Clinician Scientist Program Berlin Germany; ^3^ Department of Clinical and Biomedical Sciences, Faculty of Health and Life Sciences University of Exeter Exeter UK; ^4^ University Hospital of Pediatrics and Adolescent Medicine, Ruhr‐University Bochum Bochum Germany; ^5^ Department of Pain Medicine BG University Hospital Bergmannsheil, Ruhr‐University Bochum Bochum Germany; ^6^ Department of Neurology BG University Hospital Bergmannsheil, Ruhr‐University Bochum Bochum Germany

## Abstract

**Background:**

Mechanical detection thresholds (MDTs) are used to assess somatosensory function but can only be evaluated considering the individual sex, age and tested body region. The German Research Network on Neuropathic Pain (DFNS) provides reference values for OptiHair2, a sensitive and expensive glass filament, while nylon filaments such as the Aesthesiometer II are more durable and affordable. In this study, we compare both devices regarding their use for MDT assessment in a variety of participants, thus intending to achieve a higher range for MDT.

**Methods:**

MDTs of healthy children (< 18y), healthy adults (> 18y) and adult patients with symptoms of suspected polyneuropathy were determined using OptiHair2 glass filaments (0.25‐512mN) and Aesthesiometer II nylon filaments (0.63–235.36mN), using the method of limits according to the DFNS protocol. Testing was performed on the cheek, hand and foot in healthy participants; patients were tested on the hand and foot only. Concordance Correlation Coefficient (CCC), Pearson correlation and linear regression analysis were performed.

**Results:**

The study included 55 participants (33 female, 30 healthy adults aged 22–62 years, 10 healthy children aged 9–15 years, 15 patients aged 35–86 years). There was a relevant concordance of MDTs between Aesthesiometer II and OptiHair2 at the hand (CCC children = 0.14, healthy adults = 0.56, patients = 0.66) and foot (CCC children = 0.24, healthy adults = 0.66, patients = 0.70) but not on the cheek (CCC children = 0.00, healthy adults = 0.01).

**Conclusions:**

The Aesthesiometer II can be a viable replacement for OptiHair2 in less densely innervated areas like the feet, when screening for polyneuropathy, especially in bedside settings due to its robust materials. However, its stronger lowest force (0.63 vs. 0.25 mN) implicates poorer sensitivity for MDT assessments when testing highly innervated areas like the face.

**Significance Statement:**

This study suggests the use of robust Aesthesiometer II nylon filaments as an alternative to the more fragile OptiHair2 glass filaments for MDT assessments. While we find that only OptiHair2 seems to be suitable for more densely innervated areas like the face, Aesthesiometer II's durability and cost‐efficiency make it a useful bedside tool, especially for assessing suspected polyneuropathy in less densely innervated areas like the feet.

## 1 Introduction

Monofilaments or von Frey filaments are handheld devices with a rod usually made from glass or nylon of precise thicknesses (Rolke, Magerl, et al. 2006). They were first introduced by von Frey at the end of the 19th century (Fruhstorfer et al. 2001; von Frey 1896). Originally, monofilaments were made of human and equine hair, and their thresholds of touch recognition were based on the thickness of the hair. Later, standard sets of nylon monofilaments were introduced in the 1960s (Mamino et al. 2024; Semmes et al. 1960). Due to changes in the stiffness of plastic properties from fluctuations in temperature and humidity, the use of glass filaments was advocated (Andrews 1993; Fruhstorfer et al. 2001) and therefore recommended by the German Research Network on Neuropathic Pain (DFNS) (Rolke, Baron, et al. 2006).

Depending on the material properties of the rod, when applied to skin it will bend after exerting an exact and reproducible force. This means that multiple filaments of various thicknesses, that require different forces to be bent, can be used to determine the individuals' tactile mechanical detection threshold (MDT). The MDT tests for A‐beta fibre function, which is responsible for light touch sensation. Following the DFNS protocol, the MDT is calculated using the geometric mean between sensed and unsensed forces after the application of filaments with different thicknesses (Rolke, Magerl, et al. 2006). In simple clinical settings, monofilaments are also used to determine whether or not a patient perceives a single stimulus; for example, a ten‐gram monofilament is used for clinical screening of diabetic polyneuropathy (Perkins et al. 2010).

Assessment of MDT is typically included in protocols for Quantitative Sensory Testing (QST), which represents a standardised psychophysical procedure of sensitivity testing for the creation of a somatosensory profile (Backonja et al. 2013; Rolke, Baron, et al. 2006). For this purpose, the German Neuropathic Pain Research Network (DFNS) established a standardised test procedure, including seven tests that measure 13 different parameters, within which perception and pain thresholds are systematically determined (Rolke, Magerl, et al. 2006; Rolke, Baron, et al. 2006). To be able to compare findings across body sites and patients, the DFNS established normative values specific for body side, age decade and sex (Blankenburg et al. 2010; Magerl et al. 2010; Pfau et al. 2020; Rolke, Magerl, et al. 2006). These individualised reference values have led to a better understanding of sensory phenotypes across patient cohorts (Baron et al. 2017; Maier et al. 2010) and enabled individual assumptions about underlying mechanisms of neuropathy (Vollert et al. 2017, 2018).

However, these normative values are defined only for specific devices. In the case of MDT, the DFNS recommended device is OptiHair2 (0.25–512 mN), produced by *MRC‐Systems*, Germany (Rolke, Baron, et al. 2006). While these are considered sensitive and precise, as they are made of glass, they are relatively costly and can break if not handled carefully. These limitations make them inaccessible in low‐resource settings (Haroun et al. 2019) and unfeasible for clinical bedside testing (Reimer et al. 2020). We have previously demonstrated that in many situations, OptiHair2 can be replaced by cheaper and more robust nylon filaments like *Sorri‐Bauru* filaments (0.5–3000 mN) (Pfau et al. 2020). Unfortunately, these alternatives are not currently available, removing a practical option for bedside or low‐resource testing of mechanical detection.

Here, we compare a new set of nylon filaments, the Aesthesiometer II (*Somedic*, Sweden, (0.63–235.36 mN)) to the currently DFNS‐recommended OptiHair2 monofilaments to determine if and under which circumstances MDTs determined with each device are comparable.

## 2 Materials and Methods

### 2.1 Subjects and Setting

This prospective, randomised cross‐sectional study took place at the University Children's Hospital Bochum, Germany and the Department for Pain Medicine, BG University Hospital Bergmannsheil gGmbH, Bochum, Germany between December 2020 and March 2022. The study was approved by the ethical committee of the Ruhr‐University Bochum (register number: 20–7056, voted on 09.12.2020). Healthy subjects (age ≥ 7 years) were recruited for the study through personal contact in schools, sports clubs and by means of a study flyer. They were included in the study if eligible for QST studies by DFNS standards (Gierthmühlen et al. 2015). We used validated QST instructions for children (Blankenburg et al. 2010) and adults (Rolke, Magerl, et al. 2006).

Patients were recruited during consultation hours of the QST laboratory at BG University Hospital Bergmannsheil gGmbH. These patients were scheduled for a QST assessment within the diagnostic workup in case of suspected polyneuropathy or small fibre neuropathy. The indication for QST as part of the diagnostic work‐up for further examination of pain, dysesthesia, or paraesthesia was set by the physicians outside the study. The QST was performed based on the standardised protocol of the German Research Network on Neuropathic Pain (DFNS) independently of the study. In some cases, further diagnostics such as neurophysiological tests (NCS) or the determination of intraepidermal nerve fibre density based on a skin biopsy were carried out outside the study protocol, partly outside our hospital. The results of the additional diagnostic tests beyond QST, and especially the final evaluation, whether after the whole diagnostic work‐up polyneuropathy was confirmed or not, were not available for all and this was not part of the current study or analyses. Patients consented to the scientific use of their data from the QST.

### 2.2 Equipment

To determine the MDT, a standardised set of monofilaments of different thicknesses was used (Rolke, Baron, et al. 2006; von Frey 1896).

**FIGURE 1:**
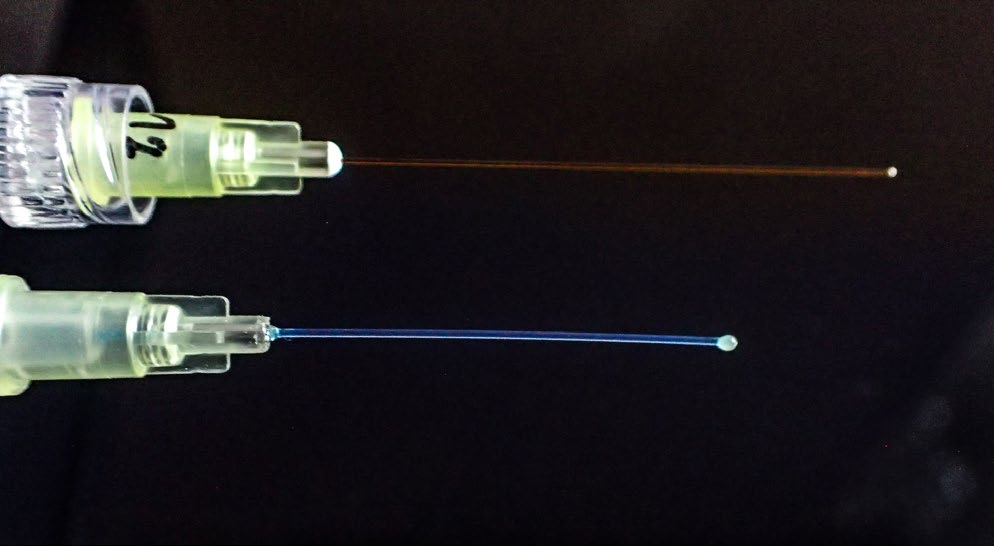
Close‐up photograph of both devices. Top: Opti Hair2, bottom: Aesthesiometer II.

The DFNS‐recommended *OptiHair 2* monofilaments set is made from optic glass fibres and consists of 12 different monofilaments, which are logarithmically scaled (0.25, 0.5, 1, 2, 4, 8, 16, 32, 64, 128, 256 and 512 mN; tolerance: ±5%), and each has a uniform rounded tip with a small epoxy pearl (diameters of 0.35–0.45 mm) (Blankenburg et al. 2010; Rolke, Magerl, et al. 2006) (Figure 1).

The Aesthesiometer II monofilaments set made from nylon contains 7 different filaments (0.63, 1.37, 3.14, 16.67, 50.0, 81.4, 235.36 mN) with a rounded ending to avoid sharp‐edged sensations (diameters range from 0.128 to 0.508 mm) (Aesthesiometer II, n.d.) (Figure 1).

### 2.3 Procedure

Each subject was asked about smoking, current pain, medications and comorbidities. In addition, intrinsic muscle reflexes and sensory thresholds were tested. In patients, the whole standardised protocol for quantitative sensory testing after the German Research Network on Neuropathic Pain (DFNS) was performed. Detailed information and assessment description can be found in Rolke, Baron, et al. (2006) (Rolke, Magerl, et al. 2006). In healthy participants, we tested the cheek, hand and foot, with the hand and cheek serving as control areas. Due to the pandemic, measurements on the cheek were no longer permitted in the patient group, so this area had to be omitted. Only the hand and foot were assessed in patients. The testing order of the sites was not randomised due to feasibility.

In healthy subjects and children only, mechanical detection thresholds, mechanical pain thresholds and vibration detection thresholds were assessed unilaterally on the subject's dominant side.

### 2.4 Mechanical Detection Thresholds (MDT)

MDT was determined according to the DFNS protocol starting with the OptiHair2 monofilaments or with the Aesthesiometer II monofilaments in randomised order at the cheek, hand and foot of the dominant side in healthy subjects and at the hand and foot in patients.

After a brief demonstration, the monofilaments were applied to the skin in a predetermined ascending or descending order at the cheek, afterwards at the hand and finally on the foot, beginning with 16 mN (Rolke et al. 2010). The person being tested was instructed to say “yes” as soon as they perceived a touch, without looking at the skin site being tested (Rolke et al. 2010). The final threshold was the geometric mean of five series of ascending and descending stimulus intensities as described in the cited literature of the DFNS framework (Baumgärtner et al. 2002). We applied increasing and decreasing von Frey filaments and recorded five points where perception changed (from “felt” to “not felt” and vice versa). The final threshold was the geometric mean of these five turning points (Baumgärtner et al. 2002). If the lightest filament was perceived, we documented half its force as the first “not perceived” value, as specified in the DFNS documentation (Rolke, Magerl, et al. 2006; Rolke et al. 2010).

### 2.5 Sample Size Calculation

This prospective study was initially designed as a non‐inferiority trial with 90 participants, equally divided between patients and healthy participants. However, due to restrictions during the COVID‐19 pandemic, the sample size was not reached. We therefore changed our analysis from a planned non‐inferiority to a concordance analysis without *p*‐values for the primary outcome.

### 2.6 Data Analysis

For data analysis, we used the logarithmically transformed mN‐values for secondary normalisation, as it has been demonstrated that MDT values are log‐normally distributed (Rolke, Magerl, et al. 2006) for the cheek (from healthy subjects), hand and foot. For patients in whom both feet were measured as part of a diagnostic QST and as the clinical symptoms were bilateral, we calculated the geometric mean of both measurements before log‐normalisation.

To perform a concordance analysis between both devices, we calculated the geometric mean and confidence intervals for OptiHair2, Aesthesiometer II and their difference, plus the Concordance Correlation Coefficients with confidence intervals. Bland–Altman plots of logarithmic values of MDT were visually presented for optical assessment of the agreement of methods.

Furthermore, we analysed correlation with Pearson's Correlation Coefficients *r* and Linear Regression using *R* ^2^, Slope and Intercept. To assess differences between all three groups with the same measurement method, the Kruskal–Wallis test was applied. For pairwise comparisons between groups (healthy paediatric vs. healthy adults, healthy adults vs. patients, healthy paediatric vs. patients), we used the Dunn test.

To visually present and assess correlation and agreement between devices, we calculated and plotted geometric means and their confidence intervals and performed and plotted linear regressions of MDT log values along with their confidence intervals. All statistical analyses and figures were produced using R (R 4.5.0 via R Studio 2024.12.1).

## 3 Results

### 3.1 Study Cohort

30 healthy adults aged 22–62 years (20 female), 10 healthy children aged 9–15 years (5 female) and 15 patients aged 35–86 years (8 female) were included in the study (Table 1).

**TABLE 1:** Basic characteristics of the population.

Characteristic	Healthy children	Healthy adults	Adult patients
Number	10	30	15
Female	5 (50%)	20 (66.67%)	8 (53.33%)
Age (median, range)	13.5 (9–15)	32 (22–62)	55 (35–86)
Height (median, range)	160 (135–180)	172.5 (159–195)	175 (163–202)
BMI (median, range)	18.7 (13–23.4)	22.1 (18.2–29.7)	26.2 (18.9–39)

Seven patients reported sensory symptoms, but no information was available regarding the final diagnosis. In four patients, skin biopsies revealed a reduced intraepidermal nerve fibre density at the thigh and/or the lower leg, but no information was available regarding the results of the nerve conduction studies. In four patients, the diagnosis of polyneuropathy was previously confirmed. Critical illness neuropathy was also subsequently diagnosed in one patient.

The healthy subjects did not report any type of pain, dysaesthesia, or complaints typical of polyneuropathy and reported no intake of any medications. MDT and VDT values were normal for all healthy individuals, compared with the DFNS reference database. In 2 adults (7%), MDTs were outside the lower cut‐off of the 95–CI of the DFNS reference values.

Patients presented to the Department of Pain Medicine for further diagnostics due to suspected polyneuropathy pain or complaints such as paraesthesia. 12 out of 15 patients indicated the presence of pain and 11 out of 15 patients were prescribed medication. Paraesthesia was reported by most of the patients (*n* = 10). Patients received a monotherapy with anticonvulsants (*n* = 4), antidepressants (*n* = 2), opioids (*n* = 1), non‐opioid analgesic drugs (*n* = 2) or a combination of anticonvulsants and non‐opioid analgesic drugs (*n* = 2) or no pain medication (*n* = 4).

In the patients' group based on the whole sensory profile, sensory loss was detected in 11 patients. Mechanical hyperalgesia was present in 6 patients, thermal hyperalgesia was present in 4 patients and both mechanical and thermal hyperalgesia were found in 2 patients.

### 3.2 Concordance Analysis With Aesthesiometer II and OptiHair2

The concordance correlation coefficient (CCC) analysis (Table 2) revealed varying degrees of agreement between OptiHair2 and Aesthesiometer II across different groups and body locations. In healthy adults, moderate agreement was observed for both the hand (CCC = 0.56, 95% CI [0.38–0.70]) and the foot (CCC = 0.66, 95% CI [0.46–0.79]), while agreement for the cheek was negligible (CCC = 0.01, 95% CI [0.00–0.02]). In healthy children, poor agreement was found across all locations, with the hand showing a CCC of 0.14 (95% CI [0.00–0.28]), the foot a CCC of 0.24 (95% CI [0.00–0.45]) and the cheek virtually no agreement (CCC = 0.00, 95% CI [0.00–0.01]). For patients, moderate agreement was observed for the hand (CCC = 0.66, 95% CI [0.26–0.86]) and the foot (CCC = 0.70, 95% CI [0.31–0.89]), indicating reasonable consistency between the two methods in these body regions.

**TABLE 2:** Geometric mean and its confidence interval and concordance correlation coefficient (CCC) of log values. Feet were assessed bilaterally for patients, and the presented values are geometric means.

		Mean [CI]^a^			CCC estimate [CI]
		OptiHair2	Aesthesiometer II	Difference	
Healthy Children	Cheek	0.48 [0.48–0.49]	0.72 [0.71–0.74]	0.40 [0.37–0.43]	0.00 [0–0.01]
	Hand	0.54 [0.5–0.59]	0.77 [0.72–0.82]	0.34 [0.28–0.40]	0.14 [0–0.28]
	Foot	0.64 [0.58–0.7]	0.84 [0.78–0.92]	0.28 [0.20–0.35]	0.24 [0–0.45]
Healthy Adults	Cheek	0.5 [0.49–0.51]	0.72 [0.71–0.73]	0.38 [0.35–0.40]	0.01 [0–0.02]
	Hand	0.69 [0.61–0.78]	0.85 [0.79–0.91]	0.21 [0.13–0.29]	0.56 [0.38–0.7]
	Foot	1 [0.9–1.2]	1.1 [0.97–1.2]	0.01 [−0.09–0.12]	0.66 [0.46–0.79]
Patients	Hand	1.2 [0.85–1.6]	1.3 [0.98–1.7]	0.08 [−0.19–0.35]	0.66 [0.26–0.86]
	Foot	2.9 [2.3–3.7]	3 [2.3–3.9]	0.02 [−0.20–0.24]	0.70 [0.31–0.89]

^a^ Geometric mean due to log‐norm values.

**FIGURE 2:**
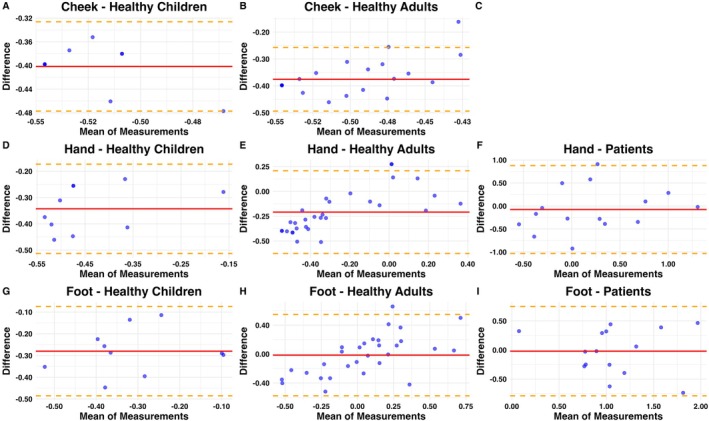
Bland–Altman plots of log‐transformed MDT values for OptiHair2 and Aesthesiometer II in the cheek (A, B), hand (D–F) and foot (G–I) in healthy children (A, D and G), healthy adults (B, E and H) and patients (F, I); no data available on patients in the cheek (C). The *x*‐axis represents the mean of the log‐transformed values, and the *y*‐axis shows the difference between the two methods. The solid red line represents the mean difference (bias), and the dashed orange lines represent the limits of agreement (mean±1.96 SD). Blue circles indicate individual measurements.

The Bland–Altman analysis (Figure 2) visually confirms that a systematic measurement difference exists for testing on the cheek, which is less prominent for testing on the hands and disappears when testing on the feet.

### 3.3 Correlation Between Aesthesiometer II and OptiHair2

**FIGURE 3:**
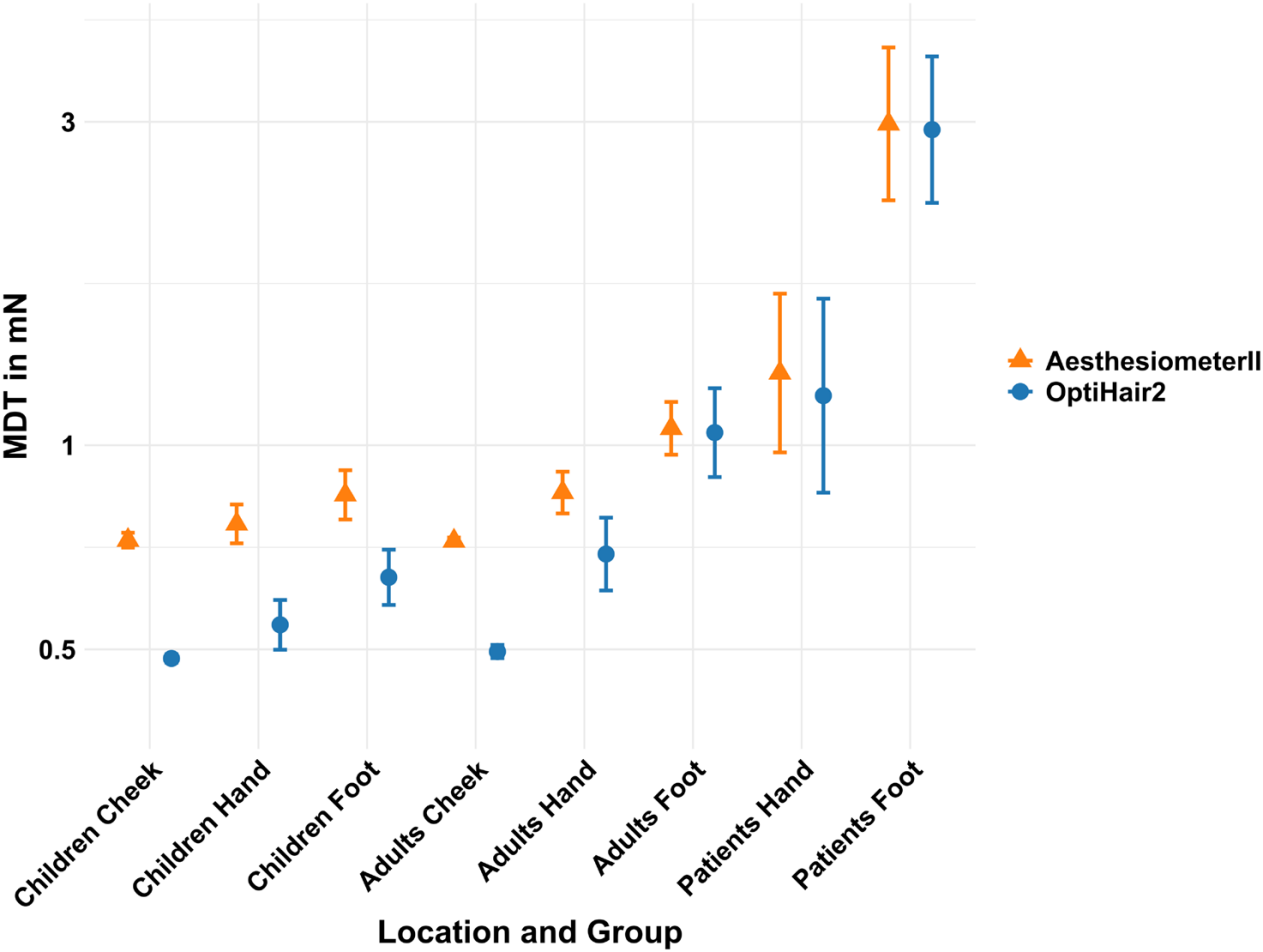
Geometric mean and confidence intervals of Opti Hair2 and Aesthesiometer II on different tested areas in healthy adults, healthy children and patients. The y‐axis is log‐transformed.

A significant correlation of MDT between Aesthesiometer II and OptiHair2 in healthy children (*r* = 0.77, *p* < 0.01; *r* = 0.74, *p* = 0.02), healthy adults (*r* = 0.83, *p* < 0.01; *r* = 0.75, *p* < 0.01) and adult patients (*r* = 0.68, *p* < 0.01; *r* = 0.70, *p* < 0.01) was noted for the hand and foot, respectively. In contrast, there was no significant correlation between the two devices in the measurement at the cheek of healthy children (*r* = 0.39, *p* = 0.27) and healthy adults (*r* = 0.32, *p* = 0.82). Patients displayed higher MDT values compared to the healthy subjects, especially at the feet (Table 3 and Figure 3). There was a significant difference when comparing the same measurement method in all three groups (healthy adults, healthy children, adult patients, *p* < 0.01) as well as when comparing the adult patients' group to either of the other groups in all tested areas (*p* < 0.01 to *p* = 0.032). A significant difference between healthy adults and healthy children was only achieved at the foot area (*p* = 0.014 for OptiHair2 and *p* = 0.043 for Aesthesiometer II) (Table S1).

**TABLE 3:** Pearson correlation and linear regression of log values. Feet were assessed bilaterally for patients, and the presented values are geometric means.

		Pearson correlation		Linear regression		
		*r*	*p*	*R* ^2^	Slope	Intercept
Healthy Children	Cheek	0.39	0.27	0.15	0.70	0.180
	Hand	0.77	< 0.01	0.59	0.60	0.099
	Foot	0.74	0.015	0.55	0.66	0.130
Healthy Adults	Cheek	0.32	0.082	0.10	0.17	−0.210
	Hand	0.83	< 0.01	0.69	0.48	0.015
	Foot	0.75	< 0.01	0.56	0.45	0.038
Patients	Hand	0.68	< 0.01	0.46	0.55	0.150
	Foot	0.70	< 0.01	0.49	0.73	0.310

**FIGURE 4:**
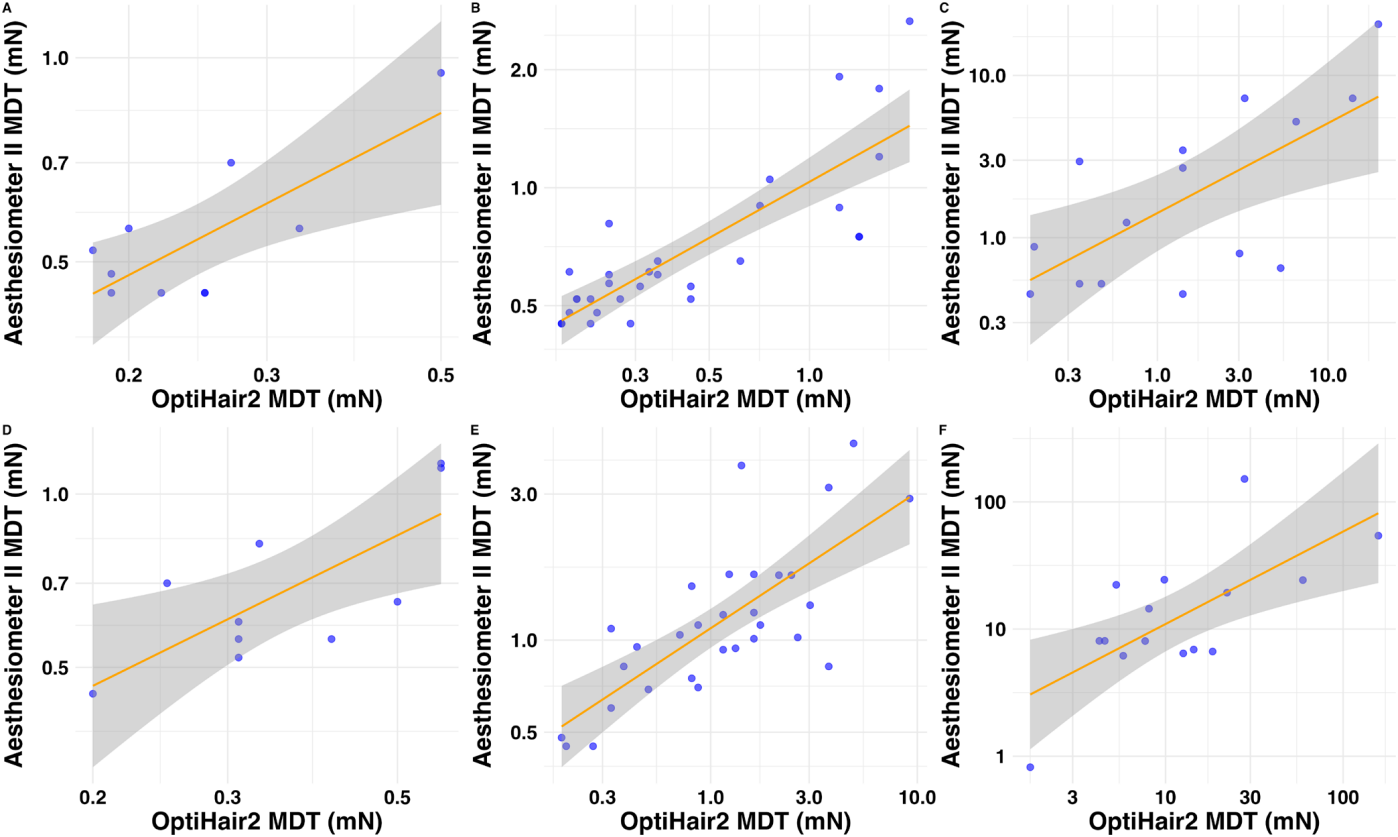
Linear regression plot of MDT values (log‐transformed, mN) for OptiHair2 and Aesthesiometer II in the hand (A–C) and the foot (D–F) across different groups: healthy children (A, D), healthy adults (B, E) and patients (C, F). The regression line (orange) is surrounded by a shaded area representing the 95% confidence interval. Blue circles represent individual measurements. Both axes are logarithmically transformed.

Regression analysis revealed significant associations between MDT measurements obtained from Opti Hair 2 and Aesthesiometer II, particularly in the hand and foot regions across all participant groups (Figure 4).

## 4 Discussion

In this study, we compare mechanical detection thresholds (MDTs) determined using glass filaments (OptiHair2) with nylon filaments (Aesthesiometer II). Our results demonstrate that, when applied to the hands or feet in adults, both devices produce results in relative agreement, with *r* values between 0.68 and 0.83. These are similar to intra‐individual reliability values previously demonstrated (Blankenburg et al. 2010; Geber et al. 2011) and likely indicate that higher agreement between methods cannot be established. Furthermore, we demonstrated a high level of absolute agreement between the two measurement methods when assessing the MDT at the hand and foot of both healthy adults and adult patients. Such findings confirm that nylon filaments may be used instead of glass filaments where these are unfeasible, while maintaining the importance of reference values produced for glass filaments.

However, when applying filaments to the highly innervated cheek in healthy subjects, there was low relative agreement and virtually no absolute agreement found between devices. This is likely a methodological limitation, resulting from the relative difference in the lightest forces of OptiHair2 (0.25mN) and Aesthesiometer II (0.63mN). This produces a floor effect: the lowest possible MDT to record with OptiHair2 is 60% lower compared to Aesthesiometer II. While this has little relevance when testing less densely innervated areas like the feet, it leads to a strong and systematic difference in highly innervated areas like the face. A similar effect was previously demonstrated with other comparable filaments (Pfau et al. 2020).

Additionally, the two devices use different increments when measuring force, making direct comparison difficult. The OptiHair2 uses twelve increments between 0.25 and 512 mN distributed evenly using a factor of 2 (Rolke, Magerl, et al. 2006). In contrast, the Aesthesiometer II has fewer increments, and these do not follow an exponential increase of force but use seven ascending increments that are not evenly spread (0.63, 1.37, 3.14, 16.67, 50, 81.4 and 235.36mN) (Aesthesiometer II, n.d.). This could explain the low absolute agreement with high relative agreement at the hand and foot, as well as the systematic bias in all the Bland–Altman plots in healthy children. Generally, due to the larger increments compared to the OptiHair2, neuropathies might be overestimated in sensitive testing areas or young populations, which are more sensitive to mechanical stimuli (Pfau et al. 2020).

Nevertheless, the use of glass filaments has limitations, namely their delicate structure and the high cost of replacing broken filaments. There is a need for alternative tools, especially for bedside sensory testing (Reimer et al. 2020; Sachau et al. 2023) and for use in resource‐limited areas (Haroun et al. 2019). Thus, nylon‐based monofilaments remain widely used despite their limitations. Examples of nylon‐based MDT measurement tools described in the literature include the bedside 0.4‐mm CMS (Chicago Medical Supply LLC, Northbrook, IL), the Touch‐Test Sensory Evaluators (North Coast Medical Inc.) (Mamino et al. 2024), the Semmes‐Weinstein von Frey Hairs (Ugo Basile) (Sieberg et al. 2024) and the Sorri‐Bauru Estesiometro filaments (Pfau et al. 2020; Reimer et al. 2020; Scholz et al. 2009; Zhu et al. 2019).

Independent of the material of the monofilaments, the shape of their tips is crucial since it defines their contact area (Rolke, Baron, et al. 2006; Rolke, Magerl, et al. 2006). The DFNS protocol specifies that filament edges should be rounded so as not to stimulate nociception (Rolke, Baron, et al. 2006; Rolke, Magerl, et al. 2006). Accordingly, Optihair2 sets use rounded tips uniform in size and shape with a 0.5 mm diameter. In contrast to other available nylon filaments like the Sorri‐Bauru Estesiometro filaments or the Ugo Basile Semmes‐Weinstein filaments, the tips of the Aesthesiometer II are likewise rounded and thus are without potentially sharp edges (Pfau et al. 2020; Sieberg et al. 2024).

Due to relevant inter‐individual differences regarding age, sex, ethnicity and different body parts, data needs to be Z‐transformed based on norm data (Rolke, Baron, et al. 2006; Rolke, Magerl, et al. 2006). This norm data is well established for OptiHair2 (Blankenburg et al. 2010; Magerl et al. 2010; Pfau et al. 2020; Rolke, Baron, et al. 2006). Currently, there is a lack of normative data for Aesthesiometer II. Without norm data for Z‐transformation, raw mN data of MDT measurement can only be interpreted intra‐individually, for example in a pre‐ and post‐intervention setup or using side‐to‐side comparison.

Generally, however, the use of these devices will depend on the sensitivity needed in the specific scenario. Nylon filaments of various forces could be used, for example to improve bedside screening of suspected polyneuropathy in specific populations. In diabetic patients, standard practice is to use a single ten‐gram monofilament (roughly a force of 100 mN). In our patient cohort, only a single patient demonstrated an MDT above this value using either Aesthesiometer II or OptiHair2, and no patient showed an MDT above 100 mN in both methods. Thus, likely none of our patients would have been identified as living with polyneuropathy if their bedside screening had been conducted with a single monofilament of ten grams. Using more sensitive nylon filaments could greatly improve diagnostic accuracy in comparison to other bedside MDT measurements, and Aesthesiometer II provides a sensible and robust option.

## 5 Limitations

Due to restrictions on research during the COVID‐19 pandemic, the sample size of *n* = 90 patients was not feasible. Due to small sample sizes in subgroups, observed CCC values come with a higher degree of uncertainty and may under‐ or overestimate true agreement, reflected in the wider confidence intervals. While this limits the precision of subgroup‐level conclusions, we are confident that despite the lowered sample size our overall results can be interpreted as valid. As another limitation, the non‐randomised testing order could lead to an order effect.

Future analyses should be based on a larger sample size and be conducted multicentrically, including an even distribution between groups for sex, age and other influencing factors, to provide reference values, which are outside the remit of this work.

## 6 Conclusion

Overall, in this preliminary study, the results suggest adequate agreement between the tools in adults and patients for hand and foot measurements, while cheek measurements consistently showed poor agreement across all groups. There seems to be a systematic bias when assessing sensitive areas such as the cheek and all areas tested in healthy children. Glass filaments of the OptiHair2, with smaller increments and lower minimum values of MDT, seem to be more sensitive to detecting elevated MDT, especially in areas with low mechanical detection thresholds. Based on this data, we cannot recommend using the Aesthesiometer II as equivalent to OptiHair2 in the DFNS protocol; however, the nylon filaments of the Aesthesiometer II can be utilised in situations where small increments and low MDT values are unnecessary, for example bedside testing at the feet for adult patients with suspected polyneuropathy. In such cases, their use could notably improve the detection rates using current tests like the single use of ten‐gram monofilaments as currently used for screening diabetic polyneuropathy (Perkins et al. 2010).

As this study was based on a limited sample from a single centre, future directions could involve adaptations of the increments of the Aesthesiometer II and multicentric development of normative data including validated Z‐scores for the Aesthesiometer II.

## Author Contributions

This study was designed by T.L., C.M., E.E.‐K. and L.E. The data was collected by A.R. and A.W. The data was analysed by J.D.W., A.R. and J.V., and the results were critically examined by all authors. J.D.W., A.R. and J.V. wrote the initial draft of the manuscript, which was edited by all authors. Figures were designed by J.D.W. and J.V.; photos were taken by C.M. All authors have approved the final version of the manuscript and agree to be accountable for all aspects of the work.

## Ethics Statement

This study highlights the use of robust Aesthesiometer II nylon filaments as an alternative to the more fragile OptiHair2 glass filaments for MDT assessments. While we find that only OptiHair2 is suited for more densely innervated areas like the face, Aesthesiometer II's durability and cost‐efficiency make it a useful bedside tool, especially for assessing suspected polyneuropathy in less densely innervated areas like the feet.

## Conflicts of Interest

The authors declare no conflicts of interest.

## Supporting information


Table S1.
