# Supplementary material for: Assessment of Mechanical Detection Thresholds in Healthy Participants and Patients With Neuropathy: A Comparison of OptiHair2 and Aesthesiometer II
Source: Eur J Pain. 2025 Jul 17;29(7):e70078. doi: 10.1002/ejp.70078 (PMC12268803; doi:10.1002/ejp.70078)
Supplement: Supplementary file 1 — Table S1. [file EJP-29-0-s001.docx]

Supplementary table 1 Differences between groups with the same measurement method

| Area | Device | Test | Statistic | p | Comparison |
| --- | --- | --- | --- | --- | --- |
| Cheek | OptiHair2 | Kruskal-Wallis | 0.258 | 0.611 |  |
| Cheek | OptiHair2 | Dunn Test |  | 0.611 | healthy adult - healthy pediatric |
| Cheek | Aesthesiometer II | Kruskal-Wallis | 0.011 | 0.914 |  |
| Cheek | Aesthesiometer II | Dunn Test |  | 0.914 | healthy adult - healthy pediatric |
| Hand | OptiHair2 | Kruskal-Wallis | 12.856 | <0.01 |  |
| Hand | OptiHair2 | Dunn Test |  | 0.087 | healthy adult - healthy pediatric |
| Hand | OptiHair2 | Dunn Test |  | 0.012 | healthy adult - patient adult |
| Hand | OptiHair2 | Dunn Test |  | <0.01 | healthy pediatric - patient adult |
| Hand | Aesthesiometer II | Kruskal-Wallis | 9.927 | <0.01 |  |
| Hand | Aesthesiometer II | Dunn Test |  | 0.11 | healthy adult - healthy pediatric |
| Hand | Aesthesiometer II | Dunn Test |  | 0.032 | healthy adult - patient adult |
| Hand | Aesthesiometer II | Dunn Test |  | <0.01 | healthy pediatric - patient adult |
| Foot | OptiHair2 | Kruskal-Wallis | 34.714 | <0.01 |  |
| Foot | OptiHair2 | Dunn Test |  | 0.014 | healthy adult - healthy pediatric |
| Foot | OptiHair2 | Dunn Test |  | <0.01 | healthy adult - patient adult |
| Foot | OptiHair2 | Dunn Test |  | <0.01 | healthy pediatric - patient adult |
| Foot | Aesthesiometer II | Kruskal-Wallis | 31.211 | <0.01 |  |
| Foot | Aesthesiometer II | Dunn Test |  | 0.043 | healthy adult - healthy pediatric |
| Foot | Aesthesiometer II | Dunn Test |  | <0.01 | healthy adult - patient adult |
| Foot | Aesthesiometer II | Dunn Test |  | <0.01 | healthy pediatric - patient adult |
